# Copy number variation in the ATP-binding cassette transporter *ABCC6* gene and *ABCC6* pseudogenes in patients with pseudoxanthoma elasticum

**DOI:** 10.1002/mgg3.137

**Published:** 2015-03-08

**Authors:** Marianne K Kringen, Camilla Stormo, Jens Petter Berg, Sharon F Terry, Christine M Vocke, Samar Rizvi, Doris Hendig, Armin P Piehler

**Affiliations:** 1 Department of Pharmacology, Oslo University HospitalUllevål, Oslo, Norway; 2 Department of Medical Biochemistry, Oslo University HospitalUllevål, Oslo, Norway; 3 Department of Medical Biochemistry, Institute of Clinical Medicine, University of OsloOslo, Norway; 4 PXE International, Genetic AllianceWashington, DC; 5 Herz- und Diabeteszentrum NRW, Institut für Laboratoriums- und Transfusionsmedizin, Universitätsklinik der Ruhr-Universität BochumBad Oeynhausen, Germany; 6 Furst Medical LaboratoryOslo, Norway

**Keywords:** *ABCC6*, copy number variation, pseudogenes, pseudoxanthoma elasticum

## Abstract

Single mutations in the ATP-binding cassette transporter (*ABCC6*) gene (OMIM 603234) are known to cause the rare autosomal recessive disease pseudoxanthoma elasticum (PXE). Recently, we have found that copy number variations (CNVs) in pseudogenes of the *ABCC6* gene are quite common. The aim of this study was to investigate the frequency and possible contribution of CNV in *ABCC6* and its pseudogenes in PXE. Genomic DNA from 212 PXE individuals were examined for copy number by pyrosequencing and quantitative polymerase chain reaction (PCR) and compared with healthy individuals. The frequency of PXE individuals with any CNV was higher than in healthy individuals. The majority of variation comprised known and possibly new deletions in the *ABCC6* gene and duplications of the *ABCC6P1* and *ABCC6P2* genes. *ABCC6* deletions and *ABCC6P2* duplications were not observed in 142 healthy individuals. In conclusion, by pyrosequencing and quantitative PCR, we were able to detect known and possibly new deletions in the *ABCC6* gene that may have caused the PXE phenotype. Pyrosequencing may be used in PXE patients who have obtained incomplete genotype from conventional techniques. The frequency of *ABCC6P2* pseudogene duplication was more common in PXE patients than healthy individuals and may affect the PXE phenotype.

## Introduction

The ATP-binding cassette transporter *ABCC6* belongs to a large family of membrane proteins (ABC transporters) that are highly conserved and present in all organisms from bacteria to man (Higgins 1992). The *ABCC6* gene is located on the short arm of chromosome 16 between two shorter, almost identical (>99% sequence identity) pseudogenes, *ABCC6P1* and *ABCC6P2* (Pulkkinen et al. 2001). Pseudogenes are generally defined as nonfunctional, meaning that they cannot produce a functional protein (Mighell et al. 2000; Balakirev and Ayala 2003). However, recently, we could show that a significant fraction of the ABC-transporter pseudogenes are transcribed. Moreover, we found evidence for a regulatory interdependency between *ABCC6* and its pseudogene *ABCC6P1* (Piehler et al. 2008).

Single mutations in *ABCC6* are known to cause the rare (prevalence between 1:25,000 and 1:100,000), autosomal recessive disease pseudoxanthoma elasticum (PXE, OMIM 264800), a metabolic disorder characterized by ectopic mineralization of soft connective tissues (Li et al. 2009; Plomp et al. 2010; Uitto et al. 2010). Patients with PXE typically present with pathological findings in the skin (yellowish papules in the flexural areas), the eye (angioid streaks and choroidal neovascularization), and the cardiovascular system (atherosclerosis). In most cases, PXE is associated with considerable morbidity and, in rare cases, mortality due to cardiovascular complications. Generally, the phenotype of PXE is highly variable showing significant inter- and intrafamilial heterogeneity (Plomp et al. 2010; Uitto et al. 2010). More than 388 mutations have been described to cause PXE (Leiden Open Variation Database at NCBI). Although an association between mutation site and severity of the disease has been postulated, no correlation has been observed (Pfendner et al. 2007; Costrop et al. 2010; Koblos et al. 2010). The wide range of the PXE phenotype severity has also led to the initiation of studies seeking modifier genes for this disease (Hendig et al. 2007; Hovnanian 2010).

In a recent study, it could be shown that larger deletions including the *ABCC6* gene significantly contribute to PXE (Costrop et al. 2010). These findings were not surprising, though, as *ABCC6* is located on chromosome 16, a known hotspot of chromosomal instability showing several genomic duplications and deletions (generally called copy number variations [CNVs]) (Sharp et al. 2006). On the basis of this information, we recently determined CNVs of *ABCC6* and *ABCC6* pseudogenes in different healthy populations, and found that CNVs of the *ABCC6* pseudogenes are quite common (Kringen et al. 2012). Having less or more copies of *ABCC6* pseudogenes is likely to influence the expression level of these pseudogenes, and therefore, may have an impact on *ABCC6* including the mRNA level, protein level and function, and the PXE phenotype. The aim of this study was to gain insight into the frequency and contribution of CNV in *ABCC6* and its pseudogenes in PXE.

## Methods

### PXE patients

DNA from patients diagnosed with PXE was obtained from the PXE International Registry and BioBank. The patients included 212 individuals and were mainly of European descent (White = 185, Hispanic = 2, Asian = 1, African American = 1, Unknown = 23). Of these, 148 (70%) were female and 64 (30%) were male. PXE phenotypes were categorized according to the five organ systems (skin, eye, gastrointestinal, vascular, and cardiac) and severity (Phenodex™, PXE International, Washington, DC) (Pfendner et al. 2007). In addition, information about diagnosis of high cholesterol or other lipid disorder was available, however, the information was self-reported and not available for all patients. *ABCC6* mutation information was available for most patients (Table S1). Written informed consent was obtained from all subjects before blood samples were taken. The study was approved by the Norwegian Regional Ethics Committees.

### Controls

Genomic DNA from healthy individuals from the National Institute of General Medical Science (NIGMS) was purchased from the Coriell Cell Repositories (Camden, NJ). The populations were Caucasians (*n* = 50), Chinese (*n* = 24), Middle East (*n* = 20), Mexicans (*n* = 24), and Africans (*n* = 24).

### CNV analysis

The CNV was analyzed in short specific regions involving exon 2, intron 7, and intron 11 of *ABCC6* (NM_001171.5; chr16: 16,243,422–16,317,328; GRCh37/hg19 Assembly). For absolute copy number determination of *ABCC6*, a TaqMan® Copy Number Assay targeting *ABCC6* specifically in intron 11 was used (Hs03952142_cn; Applied Biosystems, Foster City, CA). Two pyrosequencing assays were used to determine the relative copy number of *ABCC6P1* (NR_003569.1; chr16: 18,582,570–18,609,607; GRCh37/hg19 Assembly) versus *ABCC6* (targeting intron 7 of both genes) and the relative copy number of *ABCC6P2* (NR_023387.1; chr16: 14,916,289-14,918,559; GRCh37/hg19 Assembly) versus *ABCC6* and *ABCC6P1* (targeting exon 2 of all three genes). The analyses were performed as described previously (Kringen et al. 2012). The absolute copy number for each gene (*ABCC6*, *ABCC6P1*, and *ABCC6P2*) was finally deduced from the TaqMan® Copy Number Assay and the two pyrosequencing assays. Calculation of the absolute copy numbers of *ABCC6* pseudogenes was based on relative quantities (ratios) of the *ABCC6* gene compared to the *ABCC6* pseudogene(s). When one or more assay(s) deviated with respect to absolute copy numbers (e.g., for smaller duplications/deletions that were detectable in exon 2, but not in intron 7 or 11), the absolute copy numbers of the three genes were interpreted as the most reasonable.

The limitation of this method is that CNV is analyzed in short specific regions of *ABCC6* and *ABCC6* pseudogenes, and deletions/insertions in other parts of the genes, or other chromosomal reorganization events, may not be identified.

### Statistical methods

The Fisher's two-tailed exact test was used for testing categorical variables between patients and controls. *P*s < 0.05 were taken as statistical significance.

## Results and Discussion

Results of CNV in *ABCC6* and *ABCC6* pseudogenes were obtained from 207 of the 212 PXE individuals (161 singletons, 17 families × 2, and 4 families × 3). Five patients were excluded from further analysis because of inconclusive copy number results. Both deletions and duplications were found for *ABCC6*, *ABCC6P1*, and *ABCC6P2* in the PXE patients. The frequency of individuals with any CNV was higher than in a healthy population of Caucasian (controls) (19% and 6%, respectively; *P* = 0.02, Fisher's exact test) (PXE patients with only one member from each family compared to healthy controls; 18% and 6%, respectively; *P* = 0.045, Fisher's exact test) (Table1). The majority of variation comprised duplications of the *ABCC6P1* gene, which also have been found to be rather common in different healthy populations (Kringen et al. 2012). Duplication of the *ABCC6P2* gene was another common event that was observed in 10 PXE patients. *ABCC6P2* duplication was not observed in any of the healthy populations studied previously (Kringen et al. 2012). There are a variety of repeat elements, for example, Alu repeats, in *ABCC6* and *ABCC6* pseudogenes. Such repeats may mediate chromosomal rearrangements and have been suggested to be responsible for the existence of the *ABCC6* pseudogenes and also to have contributed to deletions in *ABCC6* causing PXE (Ringpfeil et al. 2001). The high frequency of CNV in PXE patients is in agreement with the liability of crossover events in this chromosomal area.

**Table 1:** Copy number variation in *ABCC6*, *ABCC6P1*, and *ABCC6P2* in PXE patients and healthy controls

Genes	Copies	PXE patients (*N* = 207), *N* (%)	Nonrelated PXE patients^1^ (*N* = 182), *N* (%)	Caucasians (Kringen et al. 2012) (*N* = 50), *N* (%)	Assorted populations (Kringen et al. 2012) (*N* = 142), *N* (%)
*ABCC6*	1	11 (5)	11 (6)	0 (0)	0 (0)
	2	192 (93)	168 (92)	50 (100)	140 (99)
	3	4 (2)	3 (2)	0 (0)	2 (1)
*ABCC6P1*	1	7 (4)	4 (2)	0 (0)	5 (3)
	2	186 (90)	167 (92)	47 (94)	126 (89)
	3	14 (7)	11 (6)	3 (6)	11 (8)
*ABCC6P2*	1	6 (3)	6 (3)	0 (0)	1 (1)
	2	191 (92)	167 (92)	50 (100)	141 (99)
	3	9 (4.5)	8 (4.5)	0 (0)	0 (0)
	4	1 (0.5)	1 (0.5)	0 (0)	0 (0)
Total number of individuals with any CNVs		40 (19)	33 (18)	3 (6)	19 (13)

Some PXE patients are relatives, and some patients vary in more than one gene, therefore the total number of CNVs (deviations from the normal copy of 2) exceeds the total number of individuals with CNVs. PXE, pseudoxanthoma elasticum; CNV, copy number variation.

1 Only one individual (randomly picked) from each family and singletons were included in the analysis.

In this study, we demonstrated that pyrosequencing is a fast and convenient method for the detection of CNV involving deletions of the entire or part of the *ABCC6* gene. In patients with incomplete *ABCC6* genotypes (not detected or not applicable), we identified a deletion of *ABCC6* in 10/169 (∼6%) of the uncharacterized alleles (Table S1). For 10 of the 11 analyzed PXE patients with *ABCC6* deletions, none or one mutant allele only had previously been identified. Table2 describes the predicted sizes of the observed PXE deletions: three patients had deletions in both *ABCC6* and *ABCC6P2* (#1) and three patients had deletions in *ABCC6* only (#2). Five patients had smaller deletions (#3 and #4) in *ABCC6* that were observed in either one or two of the analyzed regions (Exon 2 and/or intron 7) (Table2). These predicted deletions may represent new unidentified deletions causing PXE. One known deletion, c.179_195del is located within the sequencing region of the exon 2 – assay. This deletion was, however, not identified in our patients. The number of deletions in *ABCC6* comprised 6% of the PXE patients investigated in this study and was not observed in healthy controls (Caucasians) (Table1). Deletions in *ABCC6* comprise ∼6.5% of the genetic variation causing PXE (Costrop et al. 2010) which is in accordance with our results. Many of the deletions and duplications in *ABCC6* and *ABCC6* pseudogenes were smaller in size than found in healthy individuals. In three cases, a micro deletion (<30 kb) in *ABCC6* was accompanied by a duplication of *ABCC6P1*. This duplicated segment of *ABCC6P1* may in fact have contributed to the *ABCC6* deletion by gene conversion. Mendelian transmission of *ABCC6* pseudogene CNV could be demonstrated for a few families. In one family (three siblings) with three copies of *ABCC6P2*, the PXE manifestation was present in individuals with and without this duplication, however, eye, skin, and gastrointestinal symptoms were more severe for the two individuals with three copies of *ABCC6P2* (data not shown).

**Table 2:** Predicted deletions in *ABCC6* and nearby genes in PXE patients deduced by pyrosequencing and quantitative PCR in three specific regions: exon 2, intron 7, and intron 11

Deletion #	Chromosomal region	Minimum predicted deleted area^1^	Predicted minimum size	Genes partly or fully deleted	Number of patients with predicted deletions	Previously published deletion
1	*ABCC6P2* (ex2) *ABCC6* (ex2, int7, int11)	14,916,744–16,315,581	1.4 Mb	*ABCC6P2*, *NOMO1*, *NPIP*, *PDXDC1*, *NTAN1*, *RRN3*, *MPV17L*, *KIAA0430*, *MIR484*, *NDE1*, *MYH11*, *ABCC1*, *ABCC6*	3	Yes
2	*ABCC6* (ex2, int7, int11)	16,285,359–16,315,581	30 kb	*ABCC6*	3	Yes
3	*ABCC6* (ex2, int7)	16,297,473–16,315,581	18 kb	*ABCC6*	4	No
4	*ABCC6* (int7)	16,297,473–16,297,601	129 b	*ABCC6*	1	No

PXE, pseudoxanthoma elasticum; PCR, polymerase chain reaction.

1 Chromosome 16; GRCh37/hg19 assembly.

For the four patients with duplication in *ABCC6*, mutational information was limited (Table S1). The functional consequence of having three copies of *ABCC6* is unknown. Previously, by in vitro studies, we found that reduced mRNA expression of *ABCC6P1* could influence the mRNA expression of *ABCC6* (Piehler et al. 2008). Therefore, having three copies of *ABCC6* may similarly have an effect on *ABCC6* pseudogenes which again may affect the *ABCC6* gene expression. However, in our previous study of *ABCC6* CNVs, the expression of *ABCC6* in lymphoblastoid cell lines with two or three copies of *ABCC6* was too low to be reliably detected by RT-qPCR or pyrosequencing (Kringen et al. 2012).

When correlating CNV of *ABCC6* and *ABCC6* pseudogenes to clinical outcome (Phenodex™ and cholesterol diagnosis), we observed a higher frequency of patients with gastrointestinal bleeding (G1 or G2 according to Phenodex™) in patients with more than two copies of *ABCC6P2* (3/6) compared to patients with two or less copies of *ABCC6P2* (11/178) (*P* = 0.02, Fisher's exact test). It would be interesting to investigate this association further as the pathophysiological cause of gastrointestinal bleeding in PXE is unknown. No significant correlation was observed for other clinical phenotypes, cholesterol diagnosis, and CNV of *ABCC6* and/or *ABCC6* pseudogenes.

In summary, by pyrosequencing, we were able to detect known and possibly new deletions in the *ABCC6* gene that may have caused the PXE phenotype. This method may be used in combination with quantitative PCR in PXE patients who have obtained incomplete genotype from conventional techniques. The frequency of *ABCC6P2* pseudogene duplication was more common in PXE patients than healthy individuals and therefore may affect the PXE phenotype.

## Supporting Information

**Table S1.** Copy numbers of *ABCC6*, *ABCC6P1*, and *ABCC6P2* mutations in PXE patients.
