# Supplementary material for: Copy number variation in the ATP-binding cassette transporter ABCC6 gene and ABCC6 pseudogenes in patients with pseudoxanthoma elasticum
Source: Mol Genet Genomic Med. 2015 Mar 8;3(3):233–7. doi: 10.1002/mgg3.137 (PMC4444165; doi:10.1002/mgg3.137)
Supplement: Supplementary file 1 — Table S1. Copy numbers of ABCC6, ABCC6P1, and ABCC6P2 mutations in PXE patients. [file mgg30003-0233-sd1.docx]

**Supplementary Table 1. Copy numbers of *ABCC6*, *ABCC6P1* and *ABCC6P2* and *ABCC6* mutations in PXE patients.**

| **Our id** | ***ABCC6*_cn** | ***ABCC6P1*_cn** | ***ABCC6P2*_cn** | **Allele 1**  **DNA change; Protein change**  **(HGVS)** | **Allele 1**  **DNA change; Protein change**  **(HGVS)** | ***ABCC6*-Genotype** | **Contribution of *ABCC6*_cn on *ABCC6*-Genotype** |
| --- | --- | --- | --- | --- | --- | --- | --- |
| 1 | 2 | 2 | 2 | c.3421C>T; p.(Arg1141*) | c.1091C>G; p.(Thr364Arg) | cht |  |
| 2 | 2 | 2 | 2 | c.3421C>T; p.(Arg1141*) | c.1091C>G; p.(Thr364Arg) | cht |  |
| 4 | 2 | 3 | 2 | c.3412C>T; p.(Arg1138Trp); | c.3912delG; p.(?) | cht | - |
| 5 | 2 | 3 | 2 | c.3412C>T; p.(Arg1138Trp) | c.3912delG; p.(?) | cht | - |
| 6 | 2 | 2 | 2 | c.3421C>T; p.(Arg1141*) | Not detected | ht |  |
| 7 | 2 | 2 | 1 | c.3421C>T; p.(Arg1141*) | c.2996-1724_4209-478del; p.(?) | cht | - |
| 8 | 2 | 2 | 2 | c.1999delG; p.(fs*) | c.2996-1724_4209-478del; p.(?) | cht |  |
| 9 | 2 | 2 | 2 | c.2996-1724_4209-478del; p.(?) | c.938_939insT;p.(fs*) | cht |  |
| 10 | 2 | 2 | 2 | n/a | n/a | none |  |
| 11 | 2 | 2 | 2 | c.3421C>T; p.(Arg1141*) | Not detected | ht |  |
| 12 | 2 | 2 | 2 | n/a | n/a | none |  |
| 13 | 2 | 2 | 2 | Not detected | Not detected | none |  |
| 14 | 2 | 2 | 2 | c.3421C>T; p.(Arg1141*) | Not detected | ht |  |
| 15 | 2 | 2 | 2 | n/a | n/a | none |  |
| 16 | 2 | 2 | 2 | n/a | n/a | none |  |
| 17 | 2 | 2 | 2 | c.3490C>T; p.(Arg1164*) | Not detected | ht |  |
| 18 | 2 | 2 | 2 | c.3490C>T; p.(Arg1164*) | Not detected | ht |  |
| 19 | 2 | 2 | 2 | c.3490C>T; p.(Arg1164*) | Not detected | ht |  |
| 20 | 2 | 2 | 2 | n/a | n/a | none |  |
| 21 | 2 | 2 | 2 | n/a | n/a | none |  |
| 22 | 2 | 2 | 2 | c.3490C>T; p.(Arg1164*) | c.3490C>T; p.(Arg1164*) | hm |  |
| 23 | 2 | 2 | 2 | c.3389C>T; p.(Thr1130Met) | c.997+2_997+3delTG; p.(fs*) | cht |  |
| 24 | 2 | 2 | 2 | c.3389C>T; p.(Thr1130Met) | c.997+2_997+3delTG; p.(fs*) | cht |  |
| 25 | 2 | 2 | 2 | n/a | n/a | none |  |
| 26 | 2 | 2 | 2 | n/a | n/a | none |  |
| 27 | 2 | 2 | 3 | c.3421C>T; p.(Arg1141*) | c.2836C>A; p.(Leu946Ile) | cht | - |
| 28 | 2 | 2 | 2 | c.3421C>T; p.(Arg1141*) | Not detected | ht |  |
| 29 | 2 | 2 | 3 | c.3421C>T; p.(Arg1141*) | c.2836C>A; p.(Leu946Ile) | cht | - |
| 30 | 2 | 2 | 2 | n/a | n/a | none |  |
| 31 | 2 | 2 | 2 | c.3421C>T; p.(Arg1141*) | Not detected | ht |  |
| 32 | 2 | 2 | 2 | Not detected | Not detected | none |  |
| 33 | 2 | 1 | 2 | n/a | n/a | none | - |
| 34 | 2 | 2 | 2 | c.3415G>A; p.(Ala1139Thr) | Not detected | ht |  |
| 35 | 2 | 2 | 2 | c.3490C>T; p.(Arg1164*) | c.3490C>T; p.(Arg1164*) | hm |  |
| 36 | 2 | 2 | 2 | c.997+2delT; p.( ?) | c.2996-1724_4209-478del; p.(?) | cht |  |
| 37 | 2 | 2 | 2 | c.3421C>T; p.(Arg1141*) | c.2996-1724_4209-478del; p.(?) | cht |  |
| 38 | 1 | 2 | 1 | n/a | n/a | none | ht |
| 39 | 2 | 2 | 2 | c.3421C>T; p.(Arg1141*) | Not detected | ht |  |
| 40 | 2 | 2 | 2 | c.3421C>T; p.(Arg1141*) | c.2836C>A; p.(Leu946Ile) | cht |  |
| 41 | 2 | 2 | 2 | n/a | n/a | none |  |
| 42 | 2 | 1 | 2 | c.3421C>T; p.(Arg1141*) | c.2996-1724_4209-478del; p.(?) | cht | - |
| 44 | 2 | 2 | 2 | c.2996-1724_4209-478del; p.(?) | Not detected | ht |  |
| 45 | 2 | 3 | 2 | c.3421C>T; p.(Arg1141*) | c.2996-1724_4209-478del; p.(?) | cht | - |
| 46 | 2 | 3 | 2 | n/a | n/a | none | - |
| 47 | 2 | 2 | 2 | c.2278C>T; p.(Arg760Trp) | Not detected | ht |  |
| 48 | 2 | 2 | 2 | c.3413G>A; p.(Arg1138Gln) | c.3413G>A; p.(Arg1138Gln) | hm |  |
| 50 | 2 | 2 | 2 | c.3421C>T; p.(Arg1141*) | c.1144C>T; p.Arg382Trp) | cht |  |
| 51 | 2 | 2 | 2 | c.3662G>A; p.(Arg1221His) | c.3662G>A; p.(Arg1221His) | hm |  |
| 52 | 2 | 3 | 3 | n/a | n/a | none | - |
| 54 | 2 | 2 | 2 | c.1171A>G; p.(Arg391Gly) | c.2420G>A; p.(Arg807Gln) | cht |  |
| 55 | 2 | 2 | 2 | c.3421C>T; p.(Arg1141*) | c.3421C>T; p.(Arg1141*) | hm |  |
| 56 | 2 | 3 | 2 | c.3413G>A; p.(Arg1138Gln) | c.2787+1G>T; p.(?) | cht | - |
| 57 | 1 | 3 | 2 | c.3490C>T; p.(Arg1164*) | Not detected | ht | cht |
| 58 | 2 | 2 | 2 | c.3907G>C; p.(Ala1303Pro) | c.2836C>A; p.(Leu946Ile) | cht |  |
| 59 | 2 | 2 | 2 | c.177_185del9; p.(?) | c.2237_2238ins10; p.(fs*) | cht |  |
| 60 | 2 | 2 | 2 | c.3421C>T; p.(Arg1141*) | c.3421C>T; p.(Arg1141*) | hm |  |
| 61 | 2 | 2 | 2 | c.3421C>T; p.(Arg1141*) | c.3421C>T; p.(Arg1141*) | hm |  |
| 62 | 2 | 2 | 2 | c.3421C>T; p.(Arg1141*) | c.4015C>T; p.(Arg1339Cys) | cht |  |
| 63 | 2 | 2 | 2 | c.3412C>T; p.(Arg1138Trp) | Not detected | ht |  |
| 64 | 2 | 2 | 2 | c.3421C>T; p.(Arg1141*) | c.105delA; p.(Val37fs*44) | cht |  |
| 65 | 2 | 2 | 3 | c.2836C>A; p.(Leu946Ile) | c.2996-1724_4209-478del; p.(?) | cht | - |
| 66 | 2 | 2 | 2 | c.3421C>T; p.(Arg1141*) | c.3421C>T; p.(Arg1141*) | hm |  |
| 67 | 2 | 2 | 2 | c.3421C>T; p.(Arg1141*) | c.3421C>T; p.(Arg1141*) | hm |  |
| 68 | 2 | 2 | 2 | c.4041G>C; p.(Gln1347His) | c.2996-1724_4209-478del; p.(?) | cht |  |
| 69 | 2 | 2 | 2 | c.3389C>T; p.(Thr1130Met) | c.2836C>A; p.(Leu946Ile) | cht |  |
| 70 | 2 | 2 | 2 | c.2294; p.(Arg765Gln) | c.2787+1G>T; p.(?) | cht |  |
| 71 | 1 | 2 | 1 | c.3421C>T; p.(Arg1141*) | Not detected | ht | cht |
| 72 | 2 | 2 | 2 | c.3389C>T; p.(Thr1130Met) | c.2996-1724_4209-478del; p.(?) | cht |  |
| 73 | 2 | 2 | 2 | c.3421C>T; p.(Arg1141*) | Not detected | ht |  |
| 74 | 1 | 3 | 2 | c.2814C>G; p.(Tyr938*) | Not detected | ht | cht |
| 75 | 2 | 2 | 2 | c.2278C>T; p.(Arg760Trp) | Not detected | ht |  |
| 76 | 2 | 3 | 2 | c.1171A>G; p.(Arg391Gly) | c.3490C>T; p.(Arg1164*) | cht | - |
| 77 | 2 | 2 | 3 | c.3421C>T; p.(Arg1141*) | c.2996-1724_4209-478del; p.(?) | cht | - |
| 78 | 2 | 2 | 2 | c.1703C>T; p.(Phe568Ser) | Not detected | ht |  |
| 79 | 3 | 2 | 2 | c.3421C>T; p.(Arg1141*) | c.3661C>T; p.(Arg1221Cys) | cht | - |
| 80 | 3 | 2 | 2 | c.3421C>T; p.(Arg1141*) | c.3661C>T; p.(Arg1221Cys) | cht | - |
| 81 | 2 | 2 | 2 | c.2787+1G>T; p.(?) | Not detected | ht |  |
| 82 | 2 | 2 | 2 | c.2996-1724_4209-478del; p.(?) | c.2420G>A; p.(Arg807Gln) | cht |  |
| 83 | 2 | 2 | 2 | c.3941G>A; p.(Arg1314Gln) | c.1171A>G; p.(Arg391Gly) | cht |  |
| 84 | 2 | 2 | 2 | c.3421C>T; p.(Arg1141*) | Not detected | ht |  |
| 85 | 2 | 2 | 2 | c.3421C>T; p.(Arg1141*) | c.2996-1724_4209-478del; p.(?) | cht |  |
| 86 | 2 | 2 | 2 | c.3904G>A; p.(Gly1302Arg) | c.179_190delinsTCC; p.(Arg60_Trp64delinsLeuArg) | cht |  |
| 87 | 2 | 2 | 2 | c.3421C>T; p.(Arg1141*) | c.2836C>A; p.(Leu946Ile) | cht |  |
| 88 | 2 | 2 | 2 | c.3421C>T; p.(Arg1141*) | c.3904G>A; p.(Gly1302Arg) | cht |  |
| 89 | 2 | 2 | 2 | c.3490C>T; p.(Arg1164*) | c.2787+1G>T; p.(?) | cht |  |
| 90 | 2 | 2 | 2 | c.3421C>T; p.(Arg1141*) | c.3904G>A; p.(Gly1302Arg) | cht |  |
| 91 | 2 | 2 | 2 | c.3421C>T; p.(Arg1141*) | c.3904G>A; p.(Gly1302Arg) | cht |  |
| 92 | 2 | 2 | 2 | c.3421C>T; p.(Arg1141*) | c.3421C>T; p.(Arg1141*) | hm |  |
| 93 | 2 | 2 | 2 | n/a | n/a | none |  |
| 94 | 2 | 2 | 2 | c.3421C>T; p.(Arg1141*) | Not detected | ht |  |
| 95 | 2 | 2 | 2 | c.3421C>T; p.(Arg1141*) | c.2787+1G>T; p.(?) | cht |  |
| 96 | 2 | 3 | 2 | c.4192C>T; p.(Arg1398*) | c.2996-1724_4209-478del; p.(?) | cht | - |
| 97 | 2 | 2 | 3 | c.2996-1724_4209-478del; p.(?) | Not detected | ht | - |
| 98 | 2 | 2 | 2 | c.3421C>T; p.(Arg1141*) | Not detected | ht |  |
| 99 | 2 | 2 | 2 | c.3421C>T; p.(Arg1141*) | c.2836C>A; p.(Leu946Ile) | cht |  |
| 100 | 2 | 2 | 2 | c.2996-1724_4209-478del; p.(?) | Not detected | ht |  |
| 101 | 2 | 2 | 2 | c.3421C>T; p.(Arg1141*) | c.3421C>T; p.(Arg1141*) | hm |  |
| 102 | 2 | 2 | 2 | n/a | n/a | none |  |
| 103 | 2 | 2 | 2 | n/a | n/a | none |  |
| 104 | 2 | 2 | 2 | c.1176G>C; p.(Lys392Asn) | Not detected | ht |  |
| 105 | 2 | 1 | 2 | n/a | n/a | none | - |
| 106 | 2 | 1 | 2 | n/a | n/a | none | - |
| 107 | 2 | 1 | 2 | n/a | n/a | none | - |
| 108 | 2 | 3 | 3 | n/a | n/a | none | - |
| 109 | 2 | 2 | 2 | Not detected | Not detected | none |  |
| 110 | 2 | 2 | 2 | c.4015C>T; p.(Arg1339Cys) | c.4015C>T; p.(Arg1339Cys) | hm |  |
| 111 | 2 | 2 | 2 | c.4015C>T; p.(Arg1339Cys) | c.4015C>T; p.(Arg1339Cys) | hm |  |
| 112 | 2 | 2 | 2 | c.4015C>T; p.(Arg1339Cys) | Not detected | ht |  |
| 113 | 2 | 2 | 2 | n/a | n/a | none |  |
| 114 | 2 | 2 | 2 | Not detected | Not detected | none |  |
| 115 | 2 | 2 | 2 | c.3413G>A; p.(Arg1138Gln) | c.2304C>A; p.(Tyr768*) | cht |  |
| 116 | 2 | 2 | 2 | n/a | n/a | none |  |
| 117 | 2 | 2 | 2 | c.3421C>T; p.(Arg1141*) | Not detected | ht |  |
| 118 | 2 | 2 | 2 | c.4015C>T; p.(Arg1339Cys) | c.4015C>T; p.(Arg1339Cys) | hm |  |
| 119 | 2 | 2 | 2 | c.4015C>T; p.(Arg1339Cys) | c.4015C>T; p.(Arg1339Cys) | hm |  |
| 120 | 2 | 1 | 2 | c.2018T>C; p.(Leu673Pro) | Not detected | ht | - |
| 121 | 2 | 2 | 2 | c.4015C>T; p.(Arg1339Cys) | c.3413G>A; p.(Arg1138Gln) | cht |  |
| 122 | 2 | 2 | 2 | n/a | n/a | none |  |
| 123 | 2 | 2 | 2 | c.4015C>T; p.(Arg1339Cys) | c.3413G>A; p.(Arg1138Gln) | cht |  |
| 124 | 2 | 2 | 2 | n/a | n/a | none |  |
| 125 | 2 | 2 | 2 | c.3775delT; p.(fs*) | c.4104delC; p.(fs*) | cht |  |
| 126 | 2 | 2 | 2 | n/a | n/a | none |  |
| 127 | 2 | 2 | 2 | n/a | n/a | none |  |
| 128 | 2 | 2 | 2 | Not detected | Not detected | none |  |
| 129 | 2 | 2 | 2 | n/a | n/a | none |  |
| 130 | 2 | 2 | 3 | c.3412C>T; p.(Arg1138Trp) | c.2996-1724_4209-478del; p.(?) | cht | - |
| 131 | 2 | 2 | 2 | c.3490C>T; p.(Arg1164*) | c.2996-1724_4209-478del; p.(?) | cht |  |
| 132 | 2 | 2 | 2 | c.105delA; p.(Val37fs*44) | c.2996-1724_4209-478del; p.(?) | cht |  |
| 133 | 2 | 2 | 2 | c.2093A>C; p.(Gln698Pro) | Not detected | ht |  |
| 134 | 2 | 2 | 2 | n/a | n/a | none |  |
| 135 | 1 | 2 | 2 | c.3421C>T; p.(Arg1141*) | c.1603T>C; p.(Ser535Pro) | cht | ? |
| 136 | 2 | 2 | 2 | c.3491G>A; p.(Arg1164Gln) | c.1552C>T; p.(Arg518*) | cht |  |
| 137 | 1 | 3 | 2 | c.3421C>T; p.(Arg1141*) | Not detected | ht | cht |
| 138 | 2 | 2 | 2 | c.1132C>T; p.(Gln378*) | Not detected | ht |  |
| 139 | 2 | 2 | 2 | c.2329G>A; p.(Asp777Asn) | c.3736-1G>A; p.(?) | cht |  |
| 140 | 1 | 2 | 2 | c.3904G>A; p.(Gly1302Arg) | c.3904G>A; p.(Gly1302Arg) | hm | cht |
| 141 | 2 | 2 | 2 | Not detected | Not detected | none |  |
| 142 | 2 | 2 | 2 | Not detected | Not detected | none |  |
| 143 | 2 | 2 | 2 | c.3421C>T; p.(Arg1141*) | Not detected | ht |  |
| 144 | 2 | 2 | 2 | c.3427C>T; p.(Gln1143*) | c.3412C>T; p.(Arg1138Trp) | cht |  |
| 145 | 2 | 2 | 2 | c.3421C>T; p.(Arg1141*) | c.2996-1724_4209-478del; p.(?) | cht |  |
| 146 | 2 | 2 | 2 | c.3904G>A; p.(Gly1302Arg) | Not detected | ht |  |
| 147 | 2 | 2 | 2 | c.2836C>A; p.(Leu946Ile) | Not detected | ht |  |
| 148 | 2 | 2 | 2 | c.1087C>T; p.(Gln363*) | c.3421C>T; p.(Arg1141*) | cht |  |
| 149 | 2 | 2 | 2 | Not detected | Not detected | none |  |
| 150 | 2 | 2 | 2 | c.3412C>T; p.(Arg1138Trp) | c.4501G>A; p.(Gly1501Ser) | cht |  |
| 151 | 1 | 2 | 2 | Not detected | Not detected | none | ht |
| 152 | 2 | 2 | 2 | c.3775delT; p.(fs*) | Not detected | ht |  |
| 153 | 2 | 2 | 2 | Not detected | Not detected | none |  |
| 154 | 2 | 2 | 2 | c.2787+1G>T; p.(?) | c.3490C>T; p.(Arg1164*) | cht |  |
| 155 | 2 | 2 | 2 | c.3421C>T; p.(Arg1141*) | Not detected | ht |  |
| 157 | 2 | 2 | 2 | c.2820insC; p.(fs*) | c.3421C>T; p.(Arg1141*) | cht |  |
| 158 | 2 | 2 | 2 | n/a | n/a | none |  |
| 159 | 1 | 2 | 1 | Not detected | Not detected | none | ht |
| 160 | 2 | 2 | 2 | c.2996-1724_4209-478del; p.(?) | Not detected | ht |  |
| 161 | 2 | 2 | 2 | n/a | n/a | none |  |
| 162 | 2 | 2 | 2 | c.2831C>T; p.(Thr944Ile) | Not detected | ht |  |
| 163 | 2 | 2 | 2 | c.2294; p.(Arg765Gln) | c.2294; p.(Arg765Gln) | hm |  |
| 164 | 2 | 2 | 2 | c.2996-1724_4209-478del; p.(?) | Not detected | ht |  |
| 165 | 2 | 2 | 2 | c.1552C>T; p.(Arg518*) | c.3421C>T; p.(Arg1141*) | cht |  |
| 166 | 2 | 2 | 2 | n/a | n/a | none |  |
| 167 | 2 | 2 | 2 | c.3415G>A; p.(Ala1139Thr) | c.4335delG; p.(fs*) | cht |  |
| 168 | 2 | 2 | 2 | c.4335delG; p.(fs*) | c.4069C>T; p.(Arg1357Trp) | cht |  |
| 169 | 2 | 2 | 2 | c.1553G>A; p.(Arg518Gln) | c.3340C>T; p.(Arg1114Cys) | cht |  |
| 170 | 2 | 2 | 2 | c.1553G>A; p.(Arg518Gln) | c.2996-1724_4209-478del; p.(?) | cht |  |
| 171 | 2 | 2 | 2 | c.2996-1724_4209-478del; p.(?) | Not detected | ht |  |
| 172 | 2 | 2 | 2 | c.3421C>T; p.(Arg1141*) | c.3381G>A; p.(Met1127Ile) | cht |  |
| 173 | 2 | 2 | 2 | c.3421C>T; p.(Arg1141*) | c.4025T>C; p.(Ile1342Thr) | cht |  |
| 174 | 2 | 2 | 2 | c.3490C>T; p.(Arg1164*) | Not detected | ht |  |
| 175 | 2 | 2 | 2 | c.2252T>A; p.(Met751Lys) | Not detected | ht |  |
| 176 | 2 | 2 | 2 | Not detected | Not detected | none |  |
| 177 | 2 | 2 | 2 | Not detected | Not detected | none |  |
| 178 | 2 | 2 | 2 | c.2252T>A; p.(Met751Lys) | c.3421C>T; p.(Arg1141*) | cht |  |
| 179 | 2 | 2 | 2 | c.2787+1G>T; p.(?) | c.4016G>A; p.(Arg1339His) | cht |  |
| 180 | 2 | 2 | 2 | c.3421C>T; p.(Arg1141*) | c.4192C>T; p.(Arg1398*) | cht |  |
| 181 | 2 | 2 | 2 | Not detected | Not detected | none |  |
| 182 | 2 | 2 | 2 | c.220-1G>C; p.(?) | c.2996-1724_4209-478del; p.(?) | cht |  |
| 183 | 2 | 2 | 2 | c.3398G>C; p.(Gly1133Ala) | c.3823C>T; p.(Arg1275*) | cht |  |
| 184 | 2 | 2 | 2 | c.2996-1724_4209-478del; p.(?) | Not detected | ht |  |
| 185 | 2 | 2 | 2 | c.3141delCTC; p.(Phe1048del) | Not detected | ht |  |
| 186 | 1 | 2 | 1 | Not detected | Not detected | none | ht |
| 187 | 2 | 2 | 2 | Not detected | Not detected | none |  |
| 188 | 2 | 2 | 2 | c.3421C>T; p.(Arg1141*) | c.4016G>A; p.(Arg1339His) | cht |  |
| 189 | 2 | 2 | 2 | Not detected | Not detected | none |  |
| 190 | 2 | 2 | 1 | c.3421C>T; p.(Arg1141*) | c.3490C>T; p.(Arg1164*) | cht | - |
| 191 | 2 | 2 | 2 | n/a | n/a | none |  |
| 192 | 3 | 3 | 3 | n/a | n/a | none | - |
| 193 | 1 | 3 | 2 | Not detected | Not detected | none | ht |
| 194 | 2 | 2 | 2 | c.3421C>T; p.(Arg1141*) | c.3774_3775insC; p.(?) | cht |  |
| 195 | 2 | 2 | 2 | n/a | n/a | none |  |
| 196 | 2 | 1 | 2 | c.1484T>A; p.(Leu495His) | c.3421C>T; p.(Arg1141*) | cht | - |
| 197 | 2 | 2 | 2 | c.3490C>T; p.(Arg1164*) | Not detected | ht |  |
| 198 | 2 | 2 | 2 | c.1857_1858insC; p.(fs*) | Not detected | ht |  |
| 199 | 2 | 2 | 2 | c.2996-1724_4209-478del; p.(?) | c.3421C>T; p.(Arg1141*) | cht |  |
| 200 | 2 | 2 | 2 | c.2996-1724_4209-478del; p.(?) | Not detected | ht |  |
| 201 | 2 | 2 | 2 | Not detected | Not detected | none |  |
| 202 | 2 | 2 | 2 | n/a | n/a | none |  |
| 203 | 2 | 2 | 2 | n/a | n/a | none |  |
| 204 | 2 | 2 | 2 | n/a | n/a | none |  |
| 205 | 2 | 2 | 2 | c.3490C>T; p.(Arg1164*) | c.4004T>A; p.(Leu1335Gln) | cht |  |
| 206 | 2 | 2 | 2 | c.2093A>C; p.(Gln698Pro) | Not detected | ht |  |
| 207 | 2 | 2 | 4 | c.2996-1724_4209-478del; p.(?) | Not detected | ht | - |
| 208 | 2 | 2 | 2 | c.3421C>T; p.(Arg1141*) | Not detected | ht |  |
| 209 | 2 | 2 | 2 | c.3676C>T; p.(Leu1226Ile) | c.4448C>T; p.(Pro1483Leu) | cht |  |
| 210 | 2 | 2 | 2 | c.3421C>T; p.(Arg1141*) | c.2097G>T; p.(Glu669Asp) | cht |  |
| 211 | 3 | 2 | 2 | n/a | n/a | none | - |
| 212 | 2 | 2 | 2 | n/a | n/a | none |  |

| ht: heterozygous |
| --- |
| cht: compound-heterozygous |
| hm: homozygous  cn: copy number |
